# Gene expression profiling in *Rosa roxburghii* fruit and overexpressing *RrGGP2* in tobacco and tomato indicates the key control point of AsA biosynthesis

**DOI:** 10.3389/fpls.2022.1096493

**Published:** 2023-01-10

**Authors:** Yali Yan, Yiyi Liu, Min Lu, Chen Lu, Richard A. Ludlow, Man Yang, Wei Huang, Zeyang Liu, HuaMing An

**Affiliations:** ^1^ Engineering Research Center of National Forestry and Grassland Administration for Rosa roxburghii, Agricultural College, Guizhou University, Guiyang, China; ^2^ School of Biosciences, Cardiff University, Cardiff, United Kingdom

**Keywords:** *Rosa roxburghii* Tratt., L-ascorbic acid (AsA), fruits, overexpression, GDP-L-galactose pyrophosphatase (GGP)

## Abstract

*Rosa roxburghii* Tratt. is an important commercial horticultural crop endemic to China, which is recognized for its extremely high content of L-ascorbic acid (AsA). To understand the mechanisms underlying AsA overproduction in fruit of *R. roxburghii*, content levels, accumulation rate, and the expression of genes putatively in the biosynthesis of AsA during fruit development have been characterized. The content of AsA increased with fruit weight during development, and AsA accumulation rate was found to be highest between 60 and 90 days after anthesis (DAA), with approximately 60% of the total amount being accumulated during this period. *In vitro* incubating analysis of 70DAA fruit flesh tissues confirmed that AsA was synthesized mainly *via* the L-galactose pathway although L-Gulono-1, 4-lactone was also an effective precursor elevating AsA biosynthesis. Furthermore, in transcript level, AsA content was significantly associated with *GDP-L-galactose phosphorylase* (*RrGGP2*) gene expression. Virus-induced *RrGGP2* silencing reduced the AsA content in *R. roxburghii* fruit by 28.9%. Overexpressing *RrGGP2* increased AsA content by 8-12-fold in tobacco leaves and 2.33-3.11-fold in tomato fruit, respectively, and it showed enhanced resistance to oxidative stress caused by paraquat in transformed tobacco. These results further justified the importance of *RrGGP2* as a major control step to AsA biosynthesis in *R. roxburghii* fruit.

## Introduction

AsA (L- ascorbic acid, Vitamin C) is an essential metabolite for plants and nutrient for humans. In plants, AsA serves a range of cellular functions: as part of the antioxidant system, it scavenges free radicals produced by photosynthesis byproducts (Wang J. et al., 2013; Mitmesser et al., 2016; Akram et al., 2017) and stress reactions (Sarker and Oba, 2019; Paciolla et al., 2019; Sarker and Oba, 2020). Moreover, it functions as an enzyme cofactor and plays a crucial role in cell division and growth (Franceschi and Tarlyn, 2002), flower initiation, pathogen activity response, and metabolic gene expression regulation (Gallie, 2013; Mellidou and Kanellis, 2017; Fenech et al., 2018). AsA acts as a cofactor of 1-aminocyclopropane-1-carboxylate oxidase (ACCO), the terminal enzyme of the ethylene biosynthetic pathway, to catalyze the formation of climacteric fruit (De Tullio et al., 2004). It also serves as a starting point for producing other acids, including those found frequently in fruit like L-glyceric, L-threonic, L-oxalic, and L-tartaric acids (Debolt et al., 2007). In addition, AsA redox status is also vital in plant senescence and response to abiotic stress (Barth et al., 2004; Zhang et al., 2015). AsA is named after its ability to treat and prevent scurvy. It can scavenge free radicals and acts as an antioxidant in humans (Smirnoff, 2018) and can also reduce the risk of chronic diseases such as cancer, cardiovascular disease, iron deficiency anemia, and cataracts (Carr and Frei, 1999). Most mammals can produce ascorbic acid, but humans cannot synthesize AsA *in vivo* due to mutations in the *l-gulone-γ-lactone oxidase* (*GLOase*) gene (Chatterjee, 1973). The recommended dietary allowance (RDA) of AsA in males and females is 75 and 90 mg/day, respectively (Paciolla et al., 2019). Therefore, we must acquire AsA from plants or nutritional supplements as part of our diet to maintain and promote health. However, people in most countries, including many developed countries, cannot guarantee to meet the minimum ascorbic acid intake requirements (Linster and Van Schaftingen, 2007; Drouin et al., 2011; Yang, 2013). One important reason is that most commodity crops contain low amounts of AsA, and levels fall further due to cooking or extended postharvest storage (Lee and Kader, 2000; Grudzińska et al., 2016; Mieszczakowska-Frąc et al., 2021). Therefore, to alleviate this situation, the AsA content of crops and postharvest processing technology must be improved.

In plants, the content of AsA is maintained through synthesis, recycling and degradation. At present, there are four recognized pathways for AsA biosynthesis. The L-galactose pathway, also named the Smirnoff–Wheeler pathway, is thought to be the dominant route for AsA biosynthesis in many plants, which is supported by abundant biochemical and genetic studies (Bulley et al., 2012; Zhang et al., 2015; Bulley and Laing, 2016; Mellidou and Kanellis, 2017; Mellidou et al., 2017). The D-galacturonic pathway (Davey et al., 1999) is involved in AsA biosynthesis in strawberry (*Fragaria×ananassa*) (Cruz-Rus et al., 2011), grape (*Vitis vinifera*) (Cruz-Rus et al., 2010) and kiwifruit (*Actinidia eriantha*) (Jiang et al., 2018), by destroying cell walls to synthesize AsA. The L-gulose pathway (Wolucka and Montagu, 2003), a branch of the L-galactose pathway, starts with GDP-D-mannose, passes through several unknown enzymes, and finally passes through L-gulono-1, 4-lactone oxidase to synthesize AsA. The Myo-inositol pathway (Lorence et al., 2004) uses Myo-inositol as its precursor, which is also catalyzed by various enzymes and finally passes through L-gulono-1, 4-lactone oxidase to synthesize AsA. Meanwhile, AsA content is not only determined by synthesis, but also by recycling, in which AsA is formed by reducing oxidized forms of AsA (mono- and dehydroascorbate) (Chen et al., 2003; Yamamoto et al., 2005; Zhang Y. Y. et al., 2011; Liu F. et al., 2015; Huang et al., 2014).

*Rosa roxburghii* Tratt. is a plant in the Rosaceae family cultivated in Southwest China (Lu et al., 2021), that contains several bioactive compounds that may be beneficial to humans, including amino acids (Lin et al., 2017), flavonoids (Li et al., 2021), triterpenes (Liu et al., 2016), phenolic compounds, and especially AsA. AsA content can reach more than 2000 mg 100 g^-1^ FW, which is much higher than many fruits recognized as being rich in AsA. An et al. (2007) have described *R. roxburghii* as an ideal species to study the genetic basis of AsA production. Although Lu et al. (2016) have characterized the ascorbate metabolism genes in its leaves by genome survey sequencing, it remains unclear about the specific regulatory genes in their fruits. Previous studies on AsA synthesis in *R. roxburghii* fruit mainly focused on *Dehydroascorbate reductase* (*DHAR*), *GDP-mannose-3, 5-epimerase* (*GME*) (Huang et al., 2014) and *L-galactose dehydrogenase* (*GalLDH*) (Liu et al., 2013). Interestingly, however, the studies in *Arabidopsis* have shown that GDP-L-galactose phosphorylase (GGP), which catalyses the generation of L-galactose-1-P from GDP-L-galactose, is the first enzyme fully dedicated to AsA synthesis (Fenech et al., 2021), and its expression level is closely related to the biosynthesis of AsA in tomato (*Solanum lycopersicum.*) (Wang L. Y. et al., 2013), kiwifruit (*Actinidia* spp.) (Bulley et al., 2009) and blueberries (*Vaccinium corymbosum*) (Liu F. et al., 2015). Although the mechanism of AsA synthesis in higher plants including *R. roxburghii* has been preliminarily explored before, further genome-based research in this area will undoubtedly be more comprehensive and meaningful.

In this study, we identified for the first time that the expression level of *RrGGP2* was highly correlated with the content of AsA during fruit development based on *R. roxburghii* genome. Virus-induced *RrGGP2* silencing reduced AsA content in *R. roxburghii* fruit. *RrGGP*2 had an additive effect that enhanced AsA biosynthesis in tobacco and tomato, and the abiotic stress tolerance of transgenic tobacco was significantly improved. These results provide valuable insights into the regulatory mechanism of AsA in *R. roxburghii*.

## Materials and methods

### Plant materials and growth conditions

The *R. roxburghii* plants used in this study were six-year-old agamic trees of ‘Guinong 5’ and were grown at the Department of Horticulture, Guizhou University (26°42.408’N, 106°67.353’E). Fruits were collected every 15 days from the May 15th (15 days after anthesis, 15 DAA) until maturity (120 DAA). Fruits were frozen immediately in liquid nitrogen and stored at -80°C to measure the AsA content later and study the expression of AsA related genes.

Tomato (*Ailsa Craig*, AC) and tobacco *(Nicotiana tabacum*) plants were grown under standard greenhouse conditions as follows: 16-h day (25°C ± 2°C)/8-h night (18°C ± 2°C) cycle, PPFD, 200 ± 10 μmol m^-2^ s^-1^, and 80% relative humidity.

Transgenic T_0_ plants of tobacco were grown to maturity after selection on MS agar medium containing 0.4% Basta. T_1_ seeds were again germinated on MS agar medium with Basta and the resistant transgenic lines were grown to obtain the T_2_ homozygous lines used for further analysis.

The transformed lines of tomato were selected on a hygromycin-containing medium, and transgenic plants were confirmed *via* Polymerase Chain Reaction (PCR) and quantitative RT-PCR (qRT-PCR) using genomic DNA and cDNA from the leaves. T_2_ were used in AsA experiments.

### Plant transformation

A Full-length *GGP2* ORF (1341bp) (GeneBank accession number: HM998753) was amplified *via* PCR from *R. roxburghii* cDNA using gene-specific primers (**Supplementary Table 4**). It was inserted into a pFGC5941 vector with a CaMV35S promoter by replacing the CHSA intron (**Supplementary Figure 1A**). It was also cloned into a binary overexpression vector, pCAMBIA1301, which gets the CaMV35S promoter and NOS terminator from pBI121 (**Supplementary Figure 1B**). These vectors were introduced into *Agrobacterium tumefaciens* strain LBA4404. The constructs were subsequently transformed into Ailsa Craig (AC) tomato plants.

To generate *pTRV2-RrGGP2*, a 385bp *RrGGP2* (361-745bp) specific fragment was amplified from the cDNA of *R. roxburghii* fruit by PCR with specific primers (**Supplementary Table 4**). These primers contained an added *Xba*I and *Bam*HI restriction enzyme site at the 5′ end of forward and reverse primers, respectively. A fragment of the *RrGGP2* gene in the pMD-18T vector was excised with *Xba*I and *Bam*HI restriction enzyme and ligated with *pTRV2* after digestion with the same enzymes to yield *pTRV2-RrGGP2*. The vector was then introduced into *Agrobacterium tumefaciens* strain GV3101.

### RNA isolation and qRT-PCR

Total RNA was extracted using a TaKaRa MiniBEST Plant RNA Extraction Kit (TaKaRa, Dalian, China). cDNA was synthesized after removing the genomic DNA with the PrimeScrip RT reagent Kit with gDNA Eraser (Perfect Real Time) (TaKaRa, Dalian, China). qRT-PCR was performed on an ABI ViiA 7 DX system (Applied Biosystems) using SYBR Premix Ex Taq II (TaKaRa, Dalian, China). Transcript levels were normalized to *RrUBQ* (*ubiquitin*, internal control gene, ID: evm.model.Contig289.99) (Lu M. et al., 2020), and data analysis was performed using the 2^−ΔΔCT^ method (Livak and Schmittgen, 2001). Values for mean expression and standard deviation (SD) were calculated from the results of three independent experiments. The primer sequences used for qRT-PCR are listed in **Supplemental Table 5**.

### AsA content assays

AsA content was measured by High-Performance Liquid Chromatography (HPLC; Rizzolo et al., 1984; Yan et al., 2015). Fruit flesh (0.5g) was homogenized in 6% (w/v) metaphosphoric acid at 4 °C, then centrifuged at 16,000 × g for 20 min, and the supernatant was collected to the measurement. A C18 column (4.6 mm × 150 mm; 5 μm particle size) was used and eluted at 1 mL min^-1^ at 30 °C. A 0.2% metaphosphoric acid solution was used as the mobile phase. AsA quantification was performed at a UV absorption wavelength of 254 nm. A calibration curve was constructed using an AsA standard solution (Sigma-Aldrich) dissolved in 0.2% metaphosphoric acid, with content ranging between 4 and 400 µg ml^−1^. AsA content were quantified by the standard curve and expressed in mg per 100 g fresh weight. Three technical replicates were performed and averaged. The AsA content of tomato were measured as described by Stevens et al. (2006).

### AsA biosynthesis tested by feeding with candidate precursors

Fruits aged 70 DAA were cut into four sections of uniform size by longitudinal sectioning with a scalpel and treated with D-glucose, D-mannitol, L-galactono-1, 4-lactone, L-galactose, L-gulono-1, 4-lactone, L-gulose, Myo-inositol, D-glucuronic acid, or D-galacturonic acid, with a concentration of 15 mM for each treatment solution. The samples treated with sterile water were used as controls. All samples were incubated for 2, 4, 8, 12, 24, and 48 h at room temperature, with a natural daylight photoperiod, before being washed three times with sterile water and surface-dried on filter paper, and then were immersed in liquid nitrogen. AsA content were determined as described above.

### *Agrobacterium* inoculation of TRV-based VIGS in *R. roxburghii* fruits

The cuttage of 70DAA *R. roxburghii* fruits along with branches were cultured in a nutrient solution with controlled temperatures of 20°C/16 °C under a 16h/8h (day/night) photoperiod. The *Agrobacterium* strain GV3101 containing *pTRV2-RrGGP2* were incubated on Lennox agar supplemented with 50 mg/L kanamycin and 50 mg/L rifampicin (Sigma Aldrich) at 28°C. Osmotic buffer [10 mM MgCl_2_, 10 mM EMS (fatty acid methyl ester sulfonate), pH 5.5, 200 μM AS (acetyl eugenone)] was used to resuspended bacteria, with an OD600 of 0.8-1.2, and incubate at room temperature (25°C) without shaking for 2 h before syringe-infiltration. Approximately 300 μl of this *Agrobacterium* mixture was injected into 70DAA fruits. The fruits with good growth were then collected after 15 days. Ten fruits were subjected to each treatment, and each experiment was repeated three times.

### Physiological evaluation of transgenic plants for stress tolerance

Tobacco leaf discs or seeds of transgenic lines and WT were used to assess the tolerance to paraquatand NaCl stresses, according to Liu et al. (2013), with some modifications to the NaCl treatment. Specifically, seeds of WT and transgenic plants were surface sterilized and plated on 1/2 agar MS medium containing 150 mM NaCl.

### Chlorophyll measurement

Chlorophyll content was measured and performed as described by Liu et al. (2013).

### Sequence and phylogenetic analysis of GGP

*GGP* genes in *R. roxburghii* were identified based on the protein sequences of the GGP (VTC) family in *Arabidopsis thaliana*. Genomic information of *Arabidopsis thaliana*, *Solanum lycopersicum*, *Fragaria vesca*, *Pyrus bretschneideri*, *Mangifera indica*, *Actinidia chinensis*, *Nicotiana tabacum*, and *Malus pumila* was obtained from NCBI (https://www.ncbi.nlm.nih.gov/). Multiple sequence alignments of the protein sequences of the GGP were performed using MEGA X (7.0.14) (Kumar et al., 2018). A molecular phylogenetic tree was constructed using the neighbor-joining (NJ) method (Poisson correction and bootstrap = 1000 replicates) in MEGA X (7.0.14) (Kumar et al., 2018). TBtools (version 1.09876) software was used to characterize the exon-intron structures. The motifs of each deduced RrGGP protein were analyzed by MEME (version 4.12.012) (Bailey et al., 2009). SMART online software (http://smart.embl.de/) was used to identify domain architecture prediction.

### Statistical analysis

Data were analyzed with the Statistical Package for Social Science (SPSS) (SPSS, Chicago, Illinois, USA). Tukey’s multiple comparison tests assessed the differences among the tested lines. The level of significance was set at *P* < 0.05, and the standard deviation (SD) was calculated to analyze the sample variability.

## Results

### AsA accumulation in *R. roxburghii* fruit during development

**Figure 1:**
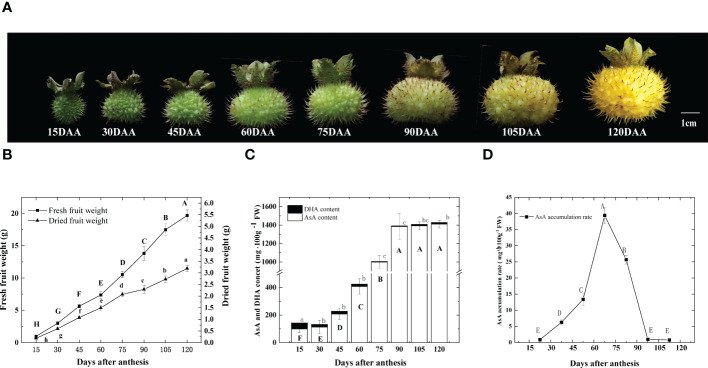
Changes of *R. roxburghii* fruits during the development. **(A)** Fruit phenotypic changes, **(B)** dynamic changes of single fruit weight, **(C)** DHA and AsA content and **(D)** the rate of AsA accumulation during the development of *R. roxburghii* fruit. Values are means of 4 replicates ± SD. AsA accumulation rate = average increase in AsA content every day, taking the median point of adjacent time as the time node. Different letters denote statistical significance.

From 15 days after anthesis (DAA), we collected fruits at eight fruit developmental stages (**Figure 1A**). As shown in **Figure 1B**, during the early stages, fruit weight increased and the dry weight held at about 20% of fresh weight, then decreased to about 15% after 75 DAA. Total ascorbic acid (T-AsA) includes AsA and DHA (L-Dehydroascorbic acid). The content of DHA remained low during the entire fruit development. The highest content was at 15 DAA, which was 42.52 mg 100 g^-1^ FW, half of the lowest AsA content recorded (**Figure 1C**). Since DHA does not contribute significantly to T-AsA, we focused on AsA in the following studies. AsA content started accumulating after anthesis, but no significant increases were observed initially. However, from 60 to 90 DAA, 976.3 mg 100g ^-1^ FW AsA was collected linearly, representing approximately 60% of the total AsA accumulation throughout the entire fruit development. From 15 DAA to 120 DAA, the AsA content varied 14.5-fold, ranging from 99.5 to 1440.2 mg 100 g^-1^ FW (**Figure 1B**), which was divided into three stages: I, an initial stage of AsA accumulation, before 60 DAA; II, a rapid stage of AsA accumulation, from 60 to 90 DAA; III, a stable stage of AsA accumulation and ripening after 90 DAA. The highest accumulation rate was 39.4 mg 100g^-1^ FW per day, which occurred between 60 and 75 DAA (**Figure 1D**). After 90 DAA, the content of AsA changed very little, as the accumulation rate decreased sharply. The 60 to 90 DAA stage appeared to be the crucial stage of AsA accumulation in the *R. roxburghii* fruit during development. As such, in the subsequent sections of this study, we focused on the period between 15 and 90 DAA.

### AsA biosynthesis analysis of *R. roxburghii* fruit

**Figure 2:**
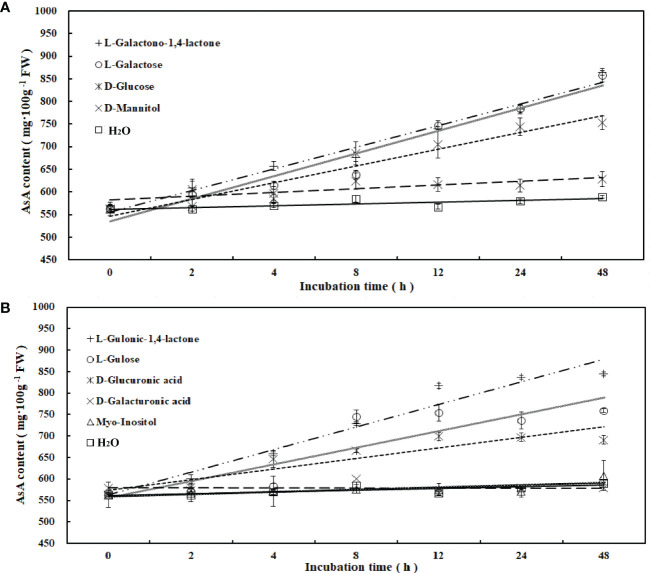
Changes of AsA content during incubation of AsA biosynthetic candidate precursors. AsA content changes following incubation of fruit flesh at 70 DAA with candidate precursors of AsA biosynthesis, at a concentrations of 15 mM. **(A, B)** included 4 and 5 candidate precursors, respectively, and water was used as a control.

To determine the main pathway of AsA synthesis in *R. roxburghii*, the fruits at 70 DAA were used for *in vitro* feeding of 9 precursors, which were intermediates in four major AsA biosynthesis pathways (**Figure 2**). Compared with the control (ddH_2_O), incubating fruit flesh on media containing exogenous putative AsA biosynthetic precursors led to significant increases in AsA content of fruit flesh. AsA accumulation increased when tissues were incubated with L-galactose, L-galactono-1, 4-lactone, L-gulonic-1, 4-lactone, D-glucuronic acid, L-gulose and D-glucose. After 48 hours, the accumulation of AsA in different medias was ranked as follows: L-galactono-1, 4-lactone (53.5%) > L -galactose (52.5%) > L-gulonic-1, 4-lactone (50.1%) > L-gulose (35%) > D-glucose (33.7%), which belonged to both the L-galactose and L-gulose pathways (**Figure 2**). Furthermore, linear regression analysis revealed that AsA content had a significant positive correlation with L-galactono-1,4-lactone (y=0.3788x+4.042, R^2^= 0.981), L-gulono-1,4-lactone (y=0.3742x+3.6162, R^2^ = 0.9561), L-galactose (y=0.392x+3.8007, R^2^ = 0.9449), D-glucose (y=0.2869x+3.9313, R^2^ = 0.9272), and L-gulose (y=0.3289x+4.3669, R^2^ = 0.7887). Contrastingly, no significant effect was observed when fruit flesh was incubated on myo-inositol (4.7%), D-Galacturonic acid (3.1%), and D-mannitol (11.7%) (**Figure 2**; **Supplemental Table 3**). These results suggested that L-galactose and L-gulose pathways might be the dominant pathways of AsA synthesis in *R. roxburghii* fruit.

### Expression of AsA biosynthetic genes in the developing fruit

**Figure 3:**
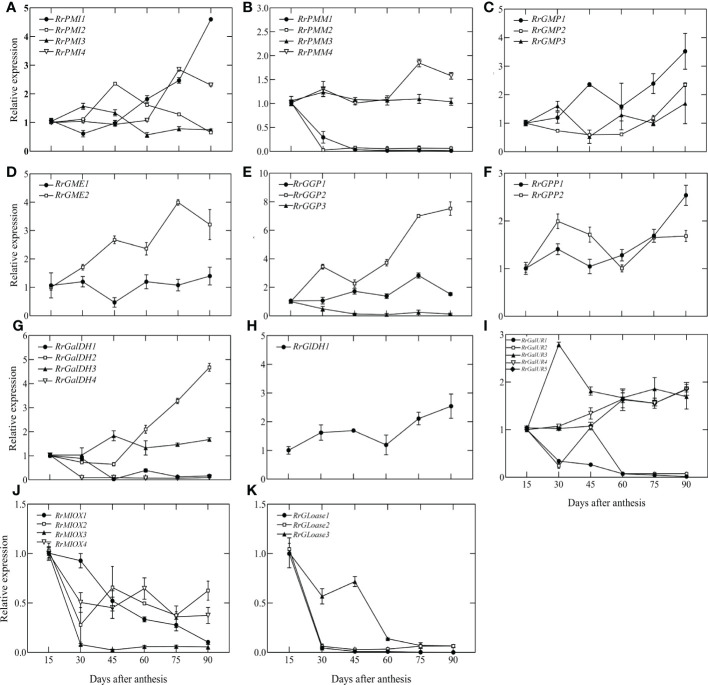
The relative expression of genes involved in AsA biosynthesis during fruit development. L-galactose pathway: **(A-H)** D-galacturonic acid pathway: **(I, H)**; Myo-inositol pathway: **(J, K)**; L-gulose pathway: **(D, K)**. For each sample, transcript levels were normalized to *UBQ*; expression over time was determined relative to a designation of ‘1’ at 15 DAA. Values are means of at least 3 replicates ± SD.

To identify potential AsA regulatory genes in biosynthetic pathways, we measured the expression levels of all genes from 15 DAA to 90 DAA by qRT-PCR, normalized to 15 DAA. The results were shown in **Figure 3**. Different members of the same gene family had different expression levels and variation trends. In the L-galactose pathway, *RrPMM1*, *RrPMM2*, *RrGGP3*, *RrGalDH1* and *RrGalDH4* were lowly expressed after 30 DAA. Compared to 15DAA, the highest expression of *RrPMI1* increased 4.6-fold, *RrGMP1* increased 3.5-fold, *RrGME2* increased 3.99-fold and *RrGalDH2* increased 4.68-fold. However, expression levels of *RrGGP2* rapidly increased at 45 DAA and peaked at 90 DAA, exhibiting a more than 7.52-fold increase. In the D-galactouronic acid pathway, the expression trend of *RrGalURs* family members differed. Among them, *RrGalUR2* and *RrGalUR5* showed a downward trend, *RrGalUR1* and *RrGalUR4* were relatively stable and *RrGalUR3* rose first and then declined. In Myo-inostiol and L-Gulose pathways, only three genes, *RrGME*, *RrMIOX* and *RrGLOase* have been identified. Both *RrMIOX* and *RrGLOase* were all decreasing in late fruit development. In particular, *RrMIOX3, RrGLOase1* and *RrGLOase2* were lowly expressed after 30 DAA. We also analyzed the correlation between all the above biosynthetic genes and AsA content in **Figure S2**. It showed that *RrGaIDH2*, *RrPMI1*, *RrGGP2* and *RrGPP1* were significantly correlated with AsA content (*P* < 0.01), while *RrMIOX* and *RrGLOase* were not. Interestingly, the above *in vitro* feeding experiments demonstrated that the L-Gulose pathway contributes to AsA synthesis (**Figure 2**). In summary, these results suggested that the L-galactose pathway may be the main pathway of AsA synthesis in *R. roxburghii* fruit, while the L-Gulose pathway may still be a secondary pathway, and *RrGGP2* was the most critical gene in the AsA biosynthesis pathway.

### Identification and phylogenetic analysis of *GGP* genes in *R. roxburghii*

**Figure 4:**
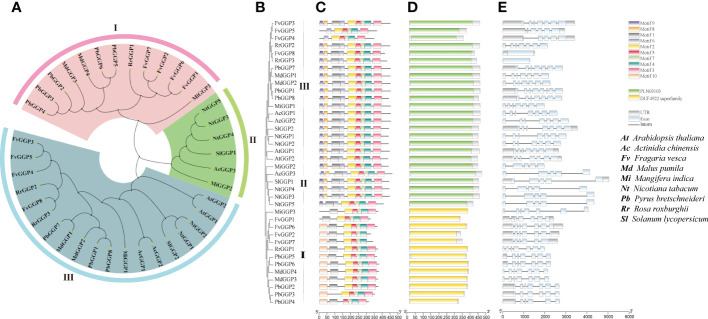
Phylogenetic relationships, gene structures and conserved protein motifs of plant *GGP* genes. **(A, B)** The un-rooted NJ phylogenetic tree of plant *GGP* genes. **(C)** Conserved motif compositions and distribution of the plant *GGP* genes. **(D)** Domain analysis of plant *GGP* genes. **(E)** Exon–intron structure of plant *GGP* genes. Phylogenetic analysis of the *GGP* genes in *Arabidopsis thaliana*, *Solanum lycopersicum*, *Fragaria vesca*, *Pyrus bretschneideri*, *Mangifera indica*, *Actinidia chinensis*, *Nicotiana tabacum*, *Malus pumila* and *Rosa roxburghii*. The unrooted tree was generated by MEGA7 using the conserved amino acid sequences of the 3 kinds RrGGP proteins. GGP protein groups were distinguished by different colors. The conserved motifs were detected using MEME software and represented by colored boxes. The length of GGP proteins can be estimated using the scale at the bottom, and the conserved motifs were shown in **Table S2**.

After analyzing gene expression levels (**Figure 3**) and examining its correlation with AsA content (**Figure S2**), we hypothesized that *RrGGP2* was a crucial gene for AsA synthesis in *R. roxburghii* fruit. We identified three GGP proteins in *R. roxburghii* using multi-sequence alignment based on the protein sequences of the GGP (VTC2 and VTC5) family in *Arabidopsis*. To investigate the evolutionary relationships among RrGGPs and GGPs of eight other representative species that had been previously studied in about AsA (*Arabidopsis thaliana*, *Solanum lycopersicum*, *Fragaria vesca*, *Pyrus bretschneideri*, *Mangifera indica*, *Actinidia chinensis*, *Nicotiana tabacum*, *Malus pumila)*, a neighbour-joining (NJ) phylogenetic tree was constructed by aligning their full-length protein sequences, with 1000 bootstrap replicates (**Figures 4A, B**; **Table S1**). All plant species had multiple GGP protein, of which strawberry has the most, with 8. The phylogenetic tree divided 38 homologs into three subgroups (I to III). Group III was the largest clade, containing 19 members, including RrGGP2 and RrGGP3, while group II was the smallest clade and consisted of only six members. Group I had 12 members, including RrGGP1. In addition, the phylogenetic analysis indicated that *GGP* genes of *R. roxburghii* were clustered with the GGP members of strawberry, suggesting a close relationship between the two Rosaceae species.

### Gene structures and conserved motifs of *GGP* genes

To reveal the structural variation of the GGP proteins in *R. roxburghii*, we further analyzed putative motifs of GGP using the program MEME (Bailey et al., 2009). The schematic distribution of these motifs among different gene groups was described (**Figure 4C**), representing their relative locations within the proteins. The multi-level consensus sequences were produced among these motifs, including those within the same group. The protein structure of the *GGP* genes corroborated with the NJ phylogenetic tree. RrGGP1 belonged to Group I, while RrGGP2 and RrGGP3 belonged to Group III. Among the ten identified motifs (**Table S2**), some were found to lack in RrGGP1 sequences compared with the other two RrGGPs proteins, with only seven motifs, motif 1, 2, 3, 4, 5, 7 and 10. However, motifs 1 to 9 were observed across the other two RrGGPs. We then performed domain analysis on these proteins using Batch CD-Search of NCBI (**Figure 4D**) (Lu et al., 2020). All the proteins in Group II and Group III contained conserved protein domain family PLN03103, a member of the superfamily cl14728, annotated as GDP-L-galactose-hexose-1-phosphate guanyltransferase (Laing et al., 2007). Proteins of Group I contained the DUF4922 superfamily, with an unknown function domain of cl14728.

As shown in **Figure 4E**, the number of exons varied significantly among different genes, ranging from 1 to 7. *FvGGP3, RrGGP2* and *RrGGP1* had the most with seven exons, and *FvGGP3* and *RrGGP2* shared similar exon-intron structures. Compared with *FvGGP3*, intron loss occurred in *FvGGP4* and *FvGGP5*. *RrGGP3* and *FvGGP8* had only one exon and exhibited no intron.

### Silencing of *RrGGP2* gene in *R. roxburghii* fruit

**Figure 5:**
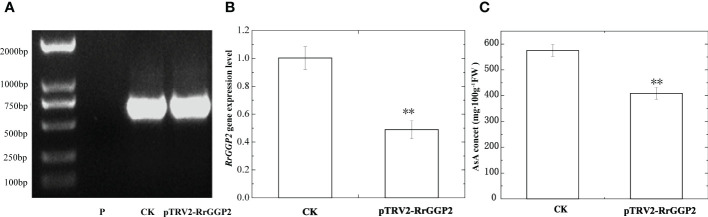
Silencing of *RrGGP2* gene in *R. roxburghii* fruits. Detection of *TRV2* transcripts **(A)**, RrGGP2 expression levels **(B)** and AsA content **(C)** after VIGS. P, untreated; CK, treated with *TRV2*; *pTRV2-RrGGP2*, treated with *pTRV2- RrGGP2*. ** Represents *P* < 0.01 compared with CK.

An effective genetic transformation system was still not available in *R. roxburghii* because of severe callus browning and challenging plant regeneration. As such, we used virus-induced gene silencing (VIGS) to study gene function. PCR was used to detect *TRV2* fragments in infected and uninfected fruits. The results were shown in **Figure 5A**, indicating that TRV virus vectors induced *RrGPP2* silencing. The qRT-PCR results showed that *pTRV2-RrGGP2* reduced the expression of *RrGGP2* by about 51.2% compared to the control (**Figure 5B**). The AsA content in the *pTRV2-RrGGP2* silenced group was decreased by 28.9% compared with the control group (**Figure 5C**). This result indicatesd that *pTRV2-RrGGP2* could reduce AsA content by silencing the endogenous *RrGGP2* gene in *R. roxburghii* fruit.

### Overexpression of *RrGGP2* in tobacco

**Figure 6:**
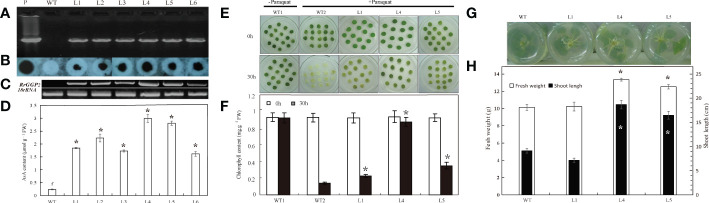
AsA content, oxidative resistance and growth status of tobacco overexpressing *RrGGP2*. **(A)** PCR analysis for the presence of the *RrGGP2* gene in Basta-resistant tobacco transgenic lines by cloning primers. **(B)** PCR-Southern dot blot analysis of the transgenic tobacco plants. Dot blot hybridization analysis following PCR products of the transgene *RrGGP2* with CaMV 35S promoter-specific primer and OCS PolyA-specific primer, where the hybridization was performed with a CaMV 35S-specific sequence as a probe. **(C)** RT-PCR analysis of total RNA from transgenic tobacco with *RrGGP2*-specific primers. **(D)** AsA content in the six transgenic lines and WT plants. Phenotypic difference **(E)** and chlorophyll content **(F)** of leaf discs after paraquat treatments for 30 hr under continuous light at 22 ± 2 °C. * Represents *P* < 0.05 compared with WT2. Rooting status after one week **(G)** and fresh weight and shoot lengths after ten weeks **(H)** of transgenic tobacco plants and control on rooting medium of 150 mM NaCl. *Represents *P* < 0.05 compared with WT, white represents fresh weight, and black represents shoot length. Lanes P*=* positive control pPFGC5941-*RrGGP2*, WT = untransformed control plants, L1–L6 = Basta-resistant T2 transgenic tobacco lines.

To investigate the role of RrGGP2 in the biosynthesis and accumulation of AsA in plants, we generated transgenic tobacco plants expressing *RrGGP2* under the control of the CaMV35S promoter to study the effect of overexpressing of exogenous *RrGGP2* on tobacco AsA content (**Figure 6D**) and resistance to oxidative stress induced by paraquat (**Figure 6E**). Six independent transgenic tobacco lines, accumulating *RrGGP2* transcripts at different levels, were generated (L1 to L6). The presence of the *RrGGP2* gene in tobacco transformants was confirmed by PCR (**Figure 6A**), and its integration into the genome was demonstrated by PCR-Southern dot blot (**Figure 6B**). The constitutive expression of *RrGGP2* in the selected transgenic lines was evaluated by RT-PCR (**Figure 6C**). The *RrGGP2* gene was expressed at different levels across the different transgenic tobacco lines. The expression of L4 was the strongest, followed by L5, while the expression of L6 was the weakest. The AsA assays performed in the six transgenic lines showed that all the transgenic lines had significantly higher AsA content than the WT plants (**Figure 6D**), which correlated with the *RrGGP2* transcript levels (**Figure 6C**). The maximum AsA content observed in L4 demonstrated 13-fold higher (2.99 μmol g^-1^ FW) compared with that in control, indicating that the overexpression of the *RrGGP2* gene in tobacco significantly increased the content of AsA in tobacco.

AsA can alleviate the oxidative effects of environmental stress on plants (Zhang et al., 2011). L1, L4 and L5 plants with high AsA content were selected to study the tolerance to paraquat induced oxidative stress. In the leaf disc assay, after 48 hours of paraquat treatment, the disc of wild-type plants showed complete senescence, while the disc of transgenic plants remained green (**Figure 6E**). The chlorophyll content of transgenic plants was significantly higher than that of WT. The chlorophyll content of L4 was 6.2-fold more elevated than WT’s (**Figure 6F**). This pattern indicated that *RrGGP2* overexpression leaded to the enhancement of antioxidant activity in transgenic lines and further alleviates the degradation of chlorophyll. Higher content of AsA can also enhance plant tolerance to salt stress (Eltayeb et al., 2011). Therefore, we treated the three transgenic lines with the highest AsA content with salt and assessed their stress tolerance. **Figure 6G** showed root growth of WT and transgenic lines after one week on a rooting medium containing 150 mM NaCl. Transgenic lines showed an apparent increase in root growth compared to WT. After ten weeks of growth, we determined the fresh weight and shoot length, and transgenic lines 4 and 5 had significantly higher fresh weight and significantly longer shoot lengths (**Figure 6H**). However, transgenic line 1 did not exhibit any salt tolerance beyond that of WT plants.

### Overexpression of *RrGGP2* in tomato

**Figure 7:**
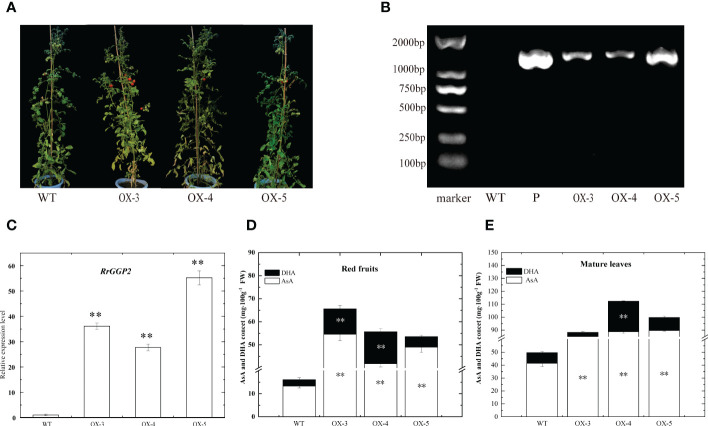
AsA content in tomato overexpressing *RrGGP2*. Transgenic lines **(A)** and confirmation of the presence of transgenes in the *RrGGP2*-overexpressing tomato lines by a PCR amplification **(B)** and qRT–PCR **(C)**. P, positive control, WT, untransformed control plants, OX-3–OX-5, Transgenic tomato plants, ** Represents p<0.01. AsA and DHA content in red fruit **(D)** and mature leaves **(E)** of RrGGP2 transgenic tomato lines. **Represents *P* < 0.01 compared with WT, white represents DHA, and black represents AsA.

To further investigate the role of *RrGGP2* in the biosynthesis and accumulation of AsA in fruit, seven *RrGGP2* over-expressing (OE) transgenic tomato plants were obtained. Three over-expressing lines (OE3, OE4 and OE5) were selected for further study (**Figure 7**). Using *R. roxburghii* fruit as the positive control (P) and *Ailsa Craig* tomato leaves as the negative control (WT). These lines were analyzed by PCR and qRT-PCR using their leaves, and the expression levels of the inserted *RrGGP2* genes in the tested transgenic lines were significantly higher compared to WT plants. However, the expression levels varied (**Figures 7B, C**).

We did not observe any change in phenotype between WT and transgenic tomato plants (**Figure 7A**). However, the content of AsA in both ripe fruit and mature leaves of over-expressing lines was significantly higher than that in WT. AsA content in over-expressing lines was increased greater in fruit (increased 3.11, 2.97 and 2.33 -fold) than in leaves (increased 0.78, 1.27 and 1.01 -fold). DHA showed the same trend in fruit, but in leaves, only the OE-4 line showed significantly improved levels (**Figures 7D, E**).

## Discussion

### AsA content in *R. roxburghii* fruit

In plants, AsA content differs significantly among species and organs. In this study, AsA in *R. roxburghii* fruit accumulated to 1440.2 mg 100 g^-1^ FW (equivalent to 81.8 µmol g ^-1^ FW) at maturity, which was far more than in other fruits, such as kiwifruit (500-1200 mg 100 g^-1^ FW, Liu et al., 2022), jujube (795.95 mg 100 g^-1^ FW, Lu et al., 2022), mango (260 mg 100 g^-1^ FW, Marialuizapa and Francom, 2008), guava(110 mg 100 g^-1^ FW, Feng et al., 2021), strawberry (50 mg 100 g^-1^ FW, Cruz-Rus et al., 2011), peach (6 mg 100 g^-1^ FW, Wang et al., 2021), apple (5 mg 100 g^-1^ FW, Li M. J. et al., 2010) and bilberry (0.6 mg 100 g^-1^ FW, Cocetta et al., 2012). Different genotypes of *R. roxburghii* also have different AsA content (Jiang et al., 2022). Our previous work showed that AsA content in fruit was much higher than in the leaves (175.6 mg 100 g^-1^ FW), flowers, and stems in *R. roxburghii* (Sun et al., 2014). The AsA content in kiwifruit and jujube accumulates rapidly in early fruit development, then decreases (Liu et al., 2022; Lu et al., 2022). However, AsA content increases throughout fruit development in most plants including tomato (Ioannidi et al., 2009), strawberry (Cruz-Rus et al., 2011) and pepper (Alós et al., 2013), which is the same in *R. roxburghii* as showed in **Figure 1C**. As *R. roxburghii* has a high AsA content, it is an effective model to study the ascorbic acid accumulation mechanism, which may provide a theoretical basis for increasing crop ascorbic acid levels more broadly and improving human nutrition.

In addition, AsA levels are also influenced by environmental factors during plant growth and development. Our previous research have clarified that different lighting conditions significantly impact AsA levels (Li, 2016; Luo et al., 2018). Adding exogenous hormones such as ABA (abscisic acid), IBA (indoleacetic acid), SA (salicylic acid) and BR (brassinolide) significantly promoted the accumulation of AsA in *R. roxburghii* (Li, 2016). Other studies have also found that the accumulation of AsA is also affected by oxidative stress (Yoshimura et al., 2014), irrigation frequency (El-Bially et al., 2018), and nitrogen fertilizer use (Smoleń and Sady, 2009; Hajaji and Gouia, 2019). At the same time, the discovery of cultivation methods to improve AsA level of *R. roxburghii* is also one of the directions of our research.

AsA participates in lutein synthesis as a cofactor of violaxanthin de-epoxidase. AsA deficiency can limit fruit coloring by reducing the activity of violaxanthin de-epoxidase *in vivo* (Müller-Moulé and Niyogi, 2002), which promotes the synthesis of lutein (Eskling et al., 1997). In this study, the results showed that AsA accumulated rapidly at 60-90 DAA. And during this stage, the fruit color gradually changed from green to yellow (**Figure 1A**). This was similar to the results in pepper, where AsA accumulated fastest when chloroplasts were transformed into chromatids during the fruit color transition from green to yellow (Alós et al., 2013). These seemed to indicate a relationship between the rapid accumulation of AsA in *R. roxburghii* fruit and the color conversion of fruit.

### AsA biosynthesis analysis in the *R. roxburghii* fruit

Feeding experiments suggested that the L-galactose and L-gulose pathways were the major pathways of AsA synthesis during *R. roxburghii* fruit ripening. Most plant species use the L-galactose pathway as their dominant pathway (Wheeler et al., 1998; Bulley and Laing, 2016). It starts from D-glucose-6-P, and AsA is generated through nine steps of enzymatic reactions (**Supplemental Figure 3**). Indeed, addition of exogenous precursors in this pathway, namely: L-galactose (increased 52.5%), L-galactono-1, 4-lactone (increased 53.5%), D-glucose (increased 33.7%), and D-mannitol (increased 11.7%), increased AsA content (**Figure 2**). From this, we deduced that *R. roxburghii* also predominantly synthesized AsA in this way.

The L-gulose pathway converts GDP-L-Gulose into AsA through L-Gulose-1-P, L-Gulose, and L-Gulono-1, 4-lactone intermediates. In our research, L-Gulono-1, 4-lactone, and L-Gulose improved AsA content (50.1% and 35%, respectively) which was consistent with those described in kiwifruit (Li M. et al., 2010). It was suggested that some plants rich in AsA synthesized AsA from the L-Gulose pathway, like animals. Previous studies show that *GLOase is* a critical control point for AsA synthesis in animals, which catalyzes the conversion of L-Gulono-1, 4-lactone to AsA (Frank et al., 1963; Smirnoff, 2001). AsA content in transgenic tobacco and tomato expressing *GLOase* from a rat accumulated up to 1.5-7 times as much AsA as the control (Jain and Nessler, 2000; Lim et al., 2012). However, in *Arabidopsis*, no significant change was found in AsA content from T-DNA insertional mutations of AT2G4676, the homologue of rat *GLOase*. *RrGLOase1, RrGLOase2* and *RrGLOase3* were all expressed downward during the AsA accumulation period of fruit, and there was no correlation with AsA content (**Figure 3** and **Supplementary Figure 2**). The genes and enzymes responsible for these additional steps in plants have not yet been identified except for *GLOase*. Therefore, the contribution of the L-gulose pathway to *R. roxburghii* needs to be further explored.

Another pathway has been reported in plants, including strawberry and citrus fruits, synthesizing AsA from D-Galacturonic acid (Cruz-Rus et al., 2011; Xu et al., 2013). Studies in kiwifruit and tomato suggest that this pathway is typically a secondary rather than a major synthetic pathway, with relatively minor contributions to total AsA (Badejo et al., 2012; Liao et al., 2021; Wang et al., 2022). In our study, adding D-galacturonic acid increased AsA content by up to 3.1%, which was not significant. The D-Galacturonic acid at the beginning of the D-galacturonate pathway is produced from the degradation of cell wall polysaccharides (Mellidou et al., 2017). Compared with strawberry and citrus fruits, the lower pectin content in *R. roxburghii* fruit (Liu Y. Q. et al., 2015) might be thereason why this pathway is especially limited here.

The Myo-inositol pathway has been extensively studied in animals, but the results in plants are inconsistent. In plants, Myo-inositol is synthesized from Glucose by L-Myo-inositol-1-phosphate synthase (MIPS) and inositol monophosphate phosphatase (IMPase). Then, Myo-inositol oxygenase (MIOX) catalyzes the transferring process of Myo-inositol into D-glucuronic acid. Next, D-glucuronic acid becomes L-Gulono-1, 4-lactone, catalyzed by something unknown. Finally, L-Gulono-1, 4-lactone is catalyzed by GLOase to become AsA. However, after we added Myo-inositol and D-glucuronic acid, there was no noticeable increase in AsA content, only 8.2% and 22.86%, respectively (**Figure 2**). Overexpression of *MIOX4* in *Arabidopsis* and tomatoes can increase AsA content (Lorence et al., 2004; Munir et al., 2020). Another investigation found out that *MIOX* did not impact AsA levels in *Arabidopsis*, but only Myo-inositol levels (Endres and Tenhaken, 2009). Coincidentally, in rice, whether the increase in abiotic stress tolerance of the strain overexpressing *OsMIOX* is directly regulated by genes or mediated by the increase in ascorbic acid content has not been proved (Duan et al., 2012). When analyzing the known genes in this pathway, we observed the low levels of *MIOXs* and a downward trend in its expression through development (**Figure 3**). Hence, the role of MIOX and other genes in the same pathway involved in AsA biosynthesis remains to be understood.

### Expression of genes related to L-galactose pathway during the *R. roxburghii* fruit development

The biosynthetic pathways of AsA in higher plants have broadly been elucidated, particularly regarding the L-galactose pathway. As per the results here, evidence suggested that biosynthesis occured predominantly through this pathway in *R. roxburghii*. Although many species use this pathway to synthesize AsA, the key rate-limiting genes of different species and organs differ. *GalDH*, *GLDH*, and *GPP* levels correlated with AsA levels in apple fruit (Li M. J. et al., 2010; Guo et al., 2011). The expression levels of *GMP*, *GME* and *GGP* in leaves were significantly higher than that in fruit, but the changing trend of these genes in leaves were not identified with the trend of AsA content (Xia et al., 2014). However, in *R. roxburghii*, consistent with the spatial localization of AsA levels, the expression of *RrGGP2* in fruit was much higher than that in leaves (Sun et al., 2014). In tomato, *GMP* (Zhang et al., 2013), *GME* (Gilbert et al., 2009; Chen et al., 2020) and *GGP* (Bulley et al., 2012) are key rate-limiting genes, and content of AsA is associated with the co-expression of the *GGP* and *GME* (Bulley et al., 2009). In kiwifruit, GGP is the bottleneck for AsA synthesis (Liu et al., 2022), as in Arabidopsis (Dowdle et al., 2007). The regulatory mechanisms in *R. roxburghii* are less well known. Consequently, here we performed qRT-PCR analysis of all the AsA synthetic pathway genes and found that their expression level only in the L-galactose pathway increased through fruit development. This trend closely matched the content of AsA (**Figure 1C**). Correlation analysis showed that *RrPMI1*, *Rr*Ga*lLDH2, RrGGP2* and *RrGPP1* were highly correlated with AsA content (*P*<0.01) (**Supplementary Figure 2**). Here, *RrGPP1* (increase 2.5-fold) contributed to AsA synthesis in the fruit of *R. roxburghii*, but a lesser degree than *RrGGP2* (increase 7.52-fold). The findings of Huang et al. in *R. roxburghii* suggested that only the gene expression and enzyme activity of *RrGME* and *RrDHAR* strongly related to AsA content (2014). However, although the *RrGME2* transcripts increased 3.99-fold, it was not very closely related to AsA content (**Supplementary Figure 2**). We therefore speculated that there may be confounding factors, as *GME* was also required for the biogenesis of cell walls and was involved in protein glycosylation (Gilbert et al., 2009).

### The key rate limiting gene for the synthesis of AsA-*RrGGP2*

GGP, as a key control step of the L-galactose pathway, is the last enzyme identified (Linster et al., 2007; Laing et al., 2007, Dowdle et al., 2007). When the *GGP* gene from kiwifruit was over-expressed in *Arabidopsis* (increased 4 fold), tomato (increased 3-6 fold) and strawberry (increased 2 fold), AsA content increased in all instances (Bulley et al., 2009; Bulley et al., 2012). Overexpressing T*riticum aestivum GGP* (*TaGGP*) in tobacco, the level of AsA in leaf tissue increased 5-fold (Broad et al., 2019). Overexpression of other genes in this pathway could also modestly increase AsA content. Overexpression of an alfalfa *GME* in Arabidopsis showed a 1.7-fold increase (Ma et al., 2014). It was also reported that *GGP* transformed into *Arabidopsis*, could increase AsA content (2.9-fold), and the transformation of the other five genes, *GMP, GME, GPP, GalDH* and *GalLDH* also increased AsA content by 1.3-, 1.4-, 1.5-, 1.2- and 1.8-fold, respectively (Zhou et al., 2012). In summary, these genes did not always have a significant effect on AsA content. However, the co-expression of *GGP* and other genes seemed to have an additive effect on the increase in AsA content. For instance, co-expression of *GGP* and *GalDH* increased AsA by 4.1-fold (Zhou et al., 2012). When *GGP* and *GME* were simultaneously inserted into *Arabidopsis*, the AsA content increased seven times (Bulley et al., 2009). Recently, in *Arabidopsis*, subcellular localizations and metabolic control analysis incorporating known kinetic characteristic can explain why other genes in the pathway can influence AsA synthesis but *GGP* is the most critical (Fenech et al., 2021).

In *R. roxburghii*, we noted that the *RrGGP2* gene expression pattern and AsA content were the same from 15DAA to 90DAA (**Figure 3**), just as in *Malus* (Davey et al., 2004). The expression of *RrGGP2* increased 7.52-fold, which was far higher than any other genes. In the *pTRV2-RrGGP2* silence group, AsA content decreased by 28.9% (**Figure 5**). Previous studies have shown that *GGP* genes may be a key rate-limiting step for AsA biosynthesis in tobacco (Laing et al., 2007) and tomato (Zhang et al., 2010). In this experiment, overexpression of *RrGGP2* gene in tobacco increased the AsA content (up to 12 times) in leaves (**Figure 6D**), which was a much greater increase than seen in our previous study, where overexpression of *RrGalLDH* in tobacco only increased AsA content by1.1-fold in leaves (Liu et al., 2013), and higher than overexpression of tomato *GGP* gene in tobacco (1.43- fold increased, Wang et al., 2014). These results all demonstrated the sizeable contribution of *RrGGP2* to the AsA production in *R. roxburghii*. In addition, over-expression of *RrDHAR* and *RrGME* in *Arabidopsis* resulted in a 3-fold and 2-fold increase in AsA content (Huang et al., 2014). Thus, GGP is most probably a rate-limiting enzyme in the L-galactose pathway in the fruit of *R. roxburghii*. In the meantime, when we overexpressed *RrGGP2* in tomato, AsA content in leaves and fruit increased by 1.27-fold and 3.11-fold, respectively (**Figure 7**), which was much higher than that in homologous transformation of *SlGGP* gene (Koukounaras et al., 2022), but lower than that of tomato overexpressing the kiwifruit *AcGGP* gene (6-fold increased, Bulley et al., 2012).As the full genome sequences of progressively more species are published, we can begin to focus on a specific key gene and other members of the gene family and analyze its mechanisms from an evolutionary perspective. Recently, it has been found that *GGP* genes exist in the whole plant kingdom, and most GGP proteins have conserved motif arrangement and composition, especially in terrestrial plants (Tao et al., 2020). We also used bioinformatic techniques to conduct whole-genome analysis on the GGP of *R. roxburghii* and 8 kinds of species that had studied AsA. Evolutionary analysis showed that *R. roxburghii* and strawberry were most closely related (**Figure 4A**). In addition, although RrGGP2 and RrGGP3 contained domain PLN03103 (**Figure 4D**), RrGGP1 contained domain DUF4922 superfamily, the functions of RrGGP1 and RrGGP3 were different from RrGGP2, due to the distant relationship of RrGGP1 (**Figure 4A**) and intron loss of *RrGGP3* (**Figure 4E**). Relatively conservative motif composition and distribution and similar exon-intron structure suggested that plant GGPs had similar catalytic functions. This corroborated the findings of Tao et al. (2020). It may also explain why *GGP* in different species can increase AsA levels when converted to tomato or other plants, but other genes in the pathway didn’t have such a conserved function.

In *R. roxburghii*, *RrGGP2* is a key rate-limiting gene for the AsA synthesis, and deserves in-depth study in many aspects. At the translation level, abundant AsA inhibits the translation of *GGP* mRNA through uORF, so the AsA content in leaves without 5’-UTR is significantly higher than that in controls (Laing et al., 2015; Zhang et al., 2018). In addition to the co-expression with other genes and feedback control of uORF at the translation level, post-translational regulation, including methylation, phosphorylation and acetylation, as well as promoter and its cis-acting elements and related transcription factors, have also been key areas of research in recent years.

### Effect of controlling AsA content to improve plant abiotic stress

The role of AsA in higher plants has been widely discussed (Conklin et al., 1997; Noctor and Foyer, 1998), especially its key impact in the ascorbic acid glutathione cycle (the main antioxidant system of plant cells). As a major antioxidant, it protects cells against the damaging effects of ROS, which are produced by normal cellular metabolism and under stress conditions (Bilska et al., 2019; Khedia et al., 2019).

Here, we overexpressed the *RrGGP2* gene in tobacco and increased the AsA content by 12-fold (**Figure 6D**). The enhanced AsA synthesis contributed to the increased tolerance of the plant to salt and paraquat stress. This is consistent with the results of Bulley et al. (2009). Furthermore, increased tolerance of transgenic plants with elevated AsA levels to salt, drought, chilling or oxidative stresses was reported with the overexpression of *PMM* (He et al., 2017), *GMP* (Lin et al., 2021), *GalLDH* (Liu et al., 2013; Dun et al., 2022), *ALO* (Bao et al., 2016), *GalUR* (Hemavathi et al., 2009; Zhang et al., 2015; Lim et al., 2016), *MIOX* (Nepal et al., 2019; Yang et al., 2021), *GLOase* (Hemavathi et al., 2010), *MDHAR* (Eltayeb et al., 2007; Haroldsen et al., 2011; Zhou et al., 2021), *DHAR* (Kwon et al., 2003; Chen and Gallie, 2006; Ushimaru et al., 2006; Qin et al., 2015; Lin et al., 2016) and *APX* (Badawi et al., 2004). In our research, transgenic lines 4 and 5 had significantly higher fresh weight and shoot length than WT when grown in the presence of salinity while transgenic line 1 did not. This may be a result of the lower accumulation rate of AsA level within transgenic line 1, which had a significantly lower total AsA content of approximately 1.8 μmol g^-1^ FW versus 3.0 μmol g^-1^ FW in line 4 (**Figure 6D**), thus suggesting a threshold of AsA content before protective effects are seen. Improving plant tolerance through increasing AsA content will alleviate the challenges brought to crops by the emergence of global extreme weather in recent years.

In summary, this is the first time that all genes in the AsA biosynthetic pathway in *R. roxburghii* are comprehensively studied concerning the whole genome. The experiment of *in vitro* incubating analysis showed that the synthesis of AsA in *R. roxburghii* was predominantly performed through the L-galactose pathway. We also analyzed the changes of AsA content and its four biosynthetic pathways gene expression levels during fruit development, the expression level of *RrGGP2* significantly correlated with AsA content. Virus-induced *RrGGP2* silencing reduced AsA level in *R. roxburghii*, and conversely, overexpression of *RrGGP2* in tobacco and tomato significantly increased AsA content. This work furthers advanced our understanding of the regulatory mechanisms controlling AsA biosynthesis and demonstrated that the *RrGGP2* was the key rate-limiting gene in *R. roxburghii*.

## Data availability statement

The original contributions presented in the study are included in the article/**Supplementary Material**. Further inquiries can be directed to the corresponding author.

## Author contributions

Conceived and designed the experiments: H-MA and ML. Performed the experiments: ML, Y-LY, Y-YL, MY, CL, WH, and Z-YL. Analyzed the data: H-MA, ML, and Y-LY. Contributed reagents/materials/analysis tools: H-MA and ML. Wrote the paper: Y-LY. Revised the paper: ML, RL, and H-MA. All authors have read and approved the final manuscript.
