# Supplementary material for: Gene expression profiling in Rosa roxburghii fruit and overexpressing RrGGP2 in tobacco and tomato indicates the key control point of AsA biosynthesis
Source: Front Plant Sci. 2023 Jan 10;13:1096493. doi: 10.3389/fpls.2022.1096493 (PMC9871823; doi:10.3389/fpls.2022.1096493)
Supplement: Supplementary file 1 [file DataSheet_1.docx]

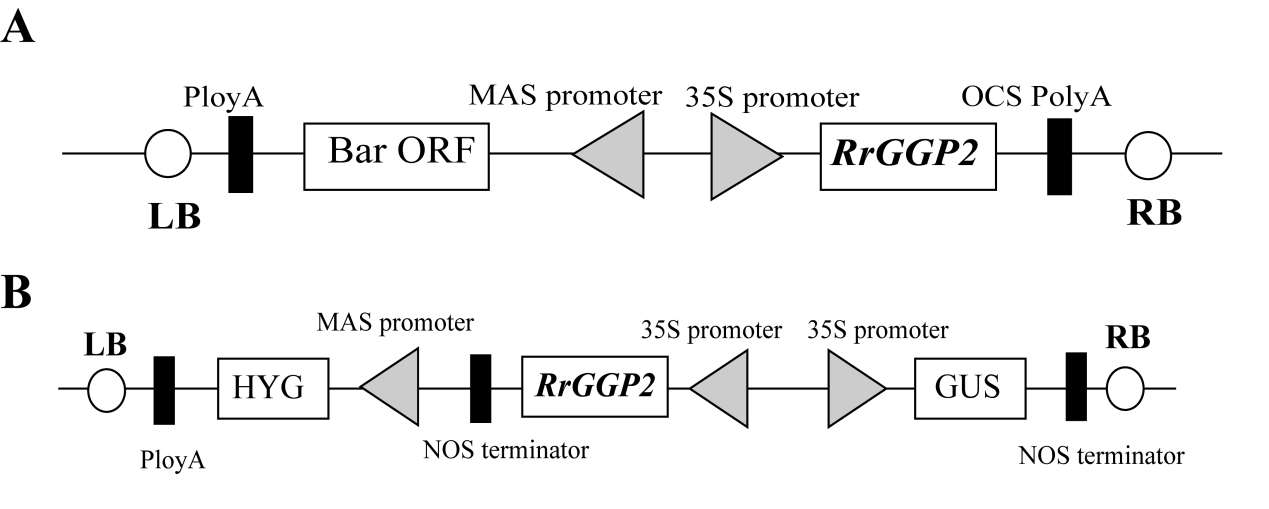


**SUPPLEMENTAL FIGURE 1 | The map of *RrGGP2* overexpression.** (A) The map of pFGC5941 for *RrGGP2* overexpression. (B) The map of Pcambia1301 for *RrGGP2* overexpression.


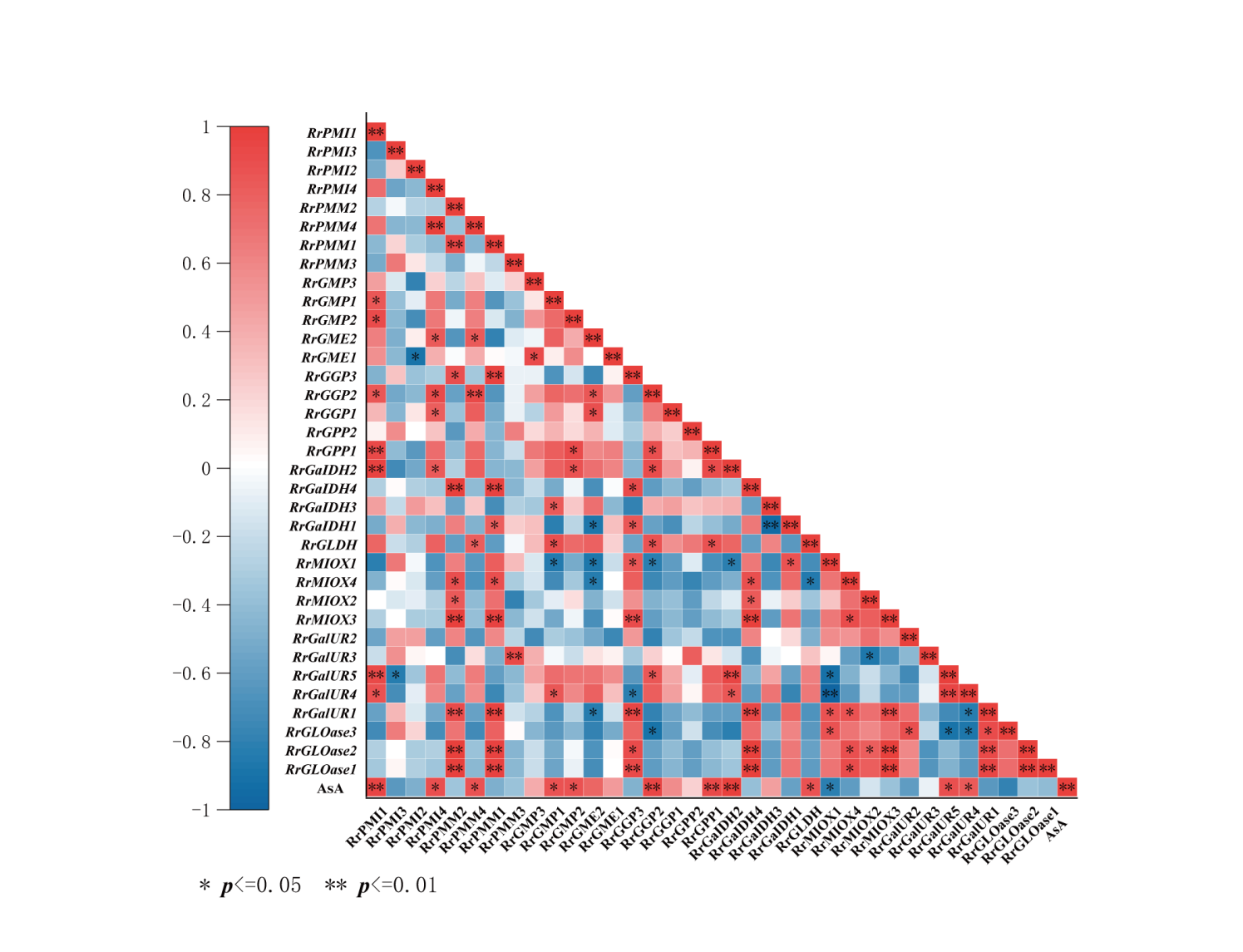


**SUPPLEMENTAL FIGURE 2 | Correlation analysis between gene expression level and AsA content.**

**
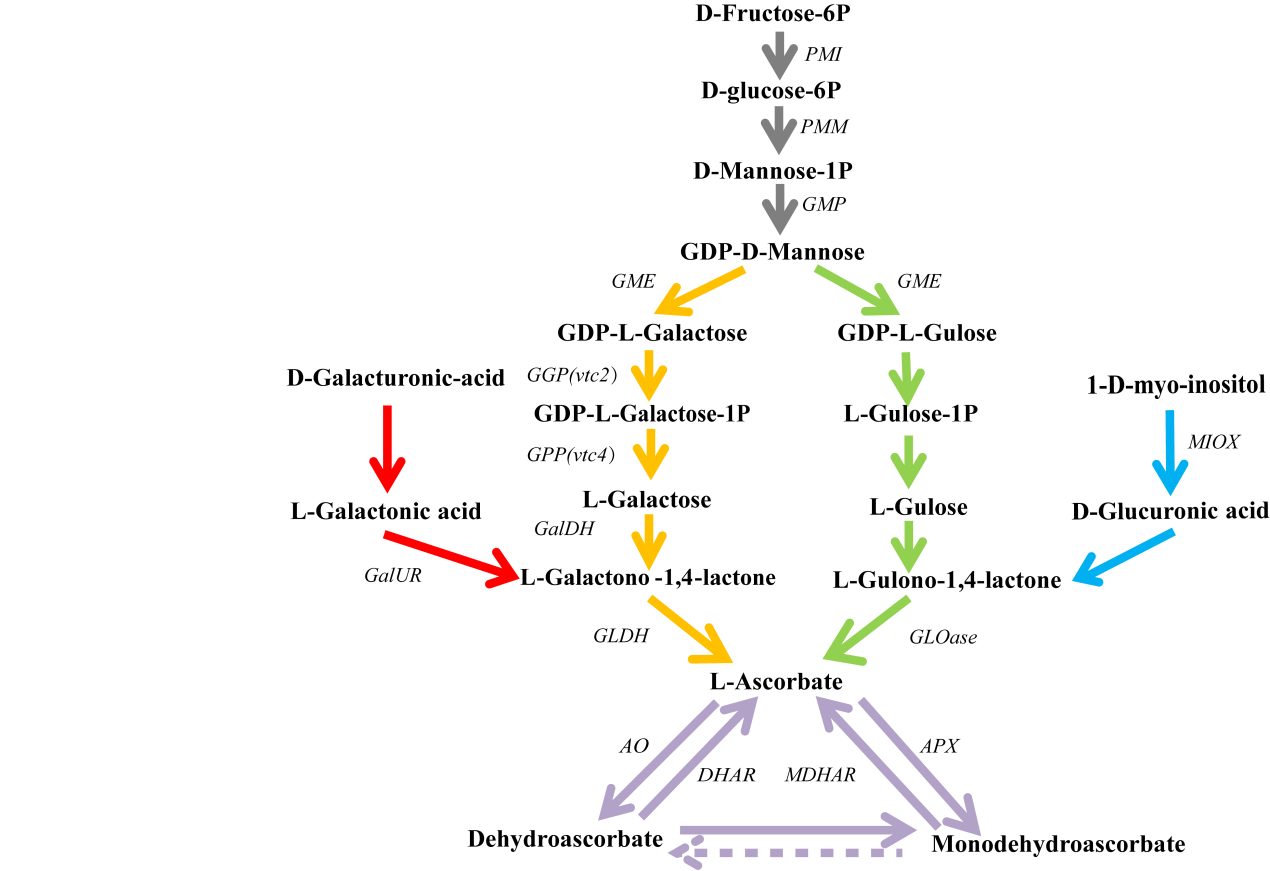
**

**SUPPLEMENTAL FIGURE 3 | AsA synthetic and recycling pathways in higher plants.** The four synthetic pathways included D-galacturonic acid pathway (red arrows), L-galactose pathway (orange arrows), L-gulose pathway (green arrows) and Myo-inositol pathway (blue arrows). Purple arrows were the recycling pathways. Arrows with dashed lines indicated undefined processes.

| **GGP gene** | **Locus gene** | **GGP gene** | **Locus gene** |
| --- | --- | --- | --- |
| AtGGP1 | At-AT4G26850.1.TAIR10 | *AcGGP1* | PSR85311.1 |
| AtGGP2 | At-AT5G55120.1.TAIR10 | *AcGGP2* | PSS06118.1 |
| FvGGP1 | Fv-FvH4_3g10190.t3.v4.0.a2 | *AcGGP3* | PSR87859.1 |
| FvGGP2 | Fv-FvH4_3g10190.t1.v4.0.a2 | *NtGGP1* | XP_016493488.1 |
| FvGGP3 | Fv-FvH4_3g23310.t1.v4.0.a2 | *NtGGP2* | XP_016507446.1 |
| FvGGP4 | Fv-FvH4_3g23310.t3.v4.0.a2 | *NtGGP3* | XP_016432651.1 |
| FvGGP5 | Fv-FvH4_3g23310.t2.v4.0.a2 | *NtGGP4* | XP_016449520.1 |
| FvGGP6 | Fv-FvH4_3g10190.t2.v4.0.a2 | *NtGGP5* | XP_016432652.1 |
| FvGGP7 | Fv-FvH4_3g10190.t4.v4.0.a2 | *MiGGP1* | XP_044501132.1 |
| FvGGP8 | Fv-FvH4_7g13380.t1.v4.0.a2 | *MiGGP2* | XP_044468497.1 |
| PbGGP1 | Pb-rna15119 | *MiGGP3* | XP_044496385.1 |
| PbGGP2 | Pb-rna16298 | *SlGGP1* | Solyc02g091510.3.1 |
| PbGGP3 | Pb-rna16297 | *SlGGP2* | Solyc06g073320.3.1 |
| PbGGP4 | Pb-rna16296 | *MdGGP1* | MD11G1211800 |
| PbGGP5 | Pb-rna45781 | *MdGGP2* | MD03G1195300 |
| PbGGP6 | Pb-rna45782 | *MdGGP3* | MD05G1272500 |
| PbGGP7 | Pb-rna50948 | *MdGGP4* | MD10G1252600 |
| PbGGP8 | Pb-rna51770 |  |  |

**SUPPLEMENTAL TABLE 1 | Statistic summary of the identified *GGP* genes.**

| **Motif ID** | **Conservative motifs** | **E-value** | **Width** | **Sites** |
| --- | --- | --- | --- | --- |
| Motif 1 | GZWEDRMQKGLFRYDVTACETKVIPGRYGFIAQLNEGRHLKKRPTEFRVD | 1.8e-1431 | 50 | 35 |
| Motif 2 | AINVSPIEYGHVLLIPRILEILPQRIDRESFLLALHMAAEAGNPYFRLGY | 1.3e-1327 | 50 | 36 |
| Motif 3 | LDTQVNPAVWEISGHMVLKRKKDYDZASEENAWKLLAEVSLSEERFQEVK | 4.1e-1261 | 50 | 36 |
| Motif 4 | NTLEDLSNVVSDACICLQENNIPYNVLISDCGKRIFLFPQC | 5.5e-1037 | 41 | 38 |
| Motif 5 | GAFATINHLHFQAYYLAVPFPVEKAPTDKI | 1.3e-796 | 30 | 38 |
| Motif 6 | VLQPFDGSKFNFTKVGQEEVLFQFEASED | 9.5e-548 | 29 | 25 |
| Motif 7 | BAGVKISELLBYPVRGLVFEG | 1.4e-434 | 21 | 38 |
| Motif 8 | GCGRNCLNKCCJPGAKLPLYAFKK | 3.7e-368 | 24 | 22 |
| Motif 9 | MLKIKRVPTVVSNYQKDEAEE | 4.4e-313 | 21 | 23 |
| Motif 10 | MVTVKQLQDDBFSSQCSPFSLQGIKIPLYRFGSNSILDDGCAGRISCVLE | 4.8e-276 | 50 | 10 |

**SUPPLEMENTAL TABLE 2 | Conserved motifs of GGP proteins.**

| **precursor** | **regression equation** | **R^2^** |
| --- | --- | --- |
| L-Galactono-1,4-lactone | y=0.3788x+4.042 | 0.981 |
| L-Gulonic-1,4-lactone | y=0.3742x+3.6162 | 0.9561 |
| L-Galactose | y=0.392x+3.8007 | 0.9449 |
| D-Glucose | y=0.2869x+3.9313 | 0.9272 |
| D-Glucuronic acid | y=0.1852x+5.1409 | 0.8365 |
| L-Gulose | y=0.3289x+4.3669 | 0.7887 |
| D-Mannitol | y=0.0741x+5.1409 | 0.6555 |
| H2O | y=0.0399x+5.5759 | 0.6395 |
| Myo-Inositol | y=0.0502x+5.1282 | 0.6055 |
| D-Galacturonic acid | y=-0.1274x+6.1066 | 0.5919 |

**SUPPLEMENTAL TABLE 3 | Linear regression analysis of precursor feeding.**

| **Gene** | **Direction** | **Primer sequence (5’–3’)** | **purpose** |
| --- | --- | --- | --- |
| RrGGP2 | F | TCGAGATGTTGAGGATCAAG | Cloning primer |
|  | R | ACCACTGATCTGCTCATTACT |  |
| RrGGP2u | F | TGCCATGGTCGAGATGTTGAGGATCAAG | Construction vector primer,Under line stands for enzyme sites. |
|  | R | GCTCTAGAACCACTGATCTGCTCATTACT |  |
| 18s | F | CAAATTTCTGCCCTATCAAC | housekeeping gene |
|  | R | CAAAATCCAACTACGAGCTT |  |
| 35S | F | GATAGTGGGATTGTGCGTCA | CaMV35S promoter |
|  | R | GCTCCTACAAATGCCATCA |  |
| P1 | F | AGCAAGTGGATTGATGTGAC | 35S Promoter |
| P2 | R | GGTAAGGATCTGAGCTACAC | OCS Ploly A |

**SUPPLEMENTAL TABLE 4 | List of primer sequences.**

|  | **Gene Name** | **Direction** | **Primer sequence (5’–3’)** |
| --- | --- | --- | --- |
| evm.model.Contig209.109 | *RrPMI1* | F | GGCTCTGAAATTGACCCGGA |
|  |  | R | TCAGAGCCCCACTTTTGGAC |
| evm.model.Contig390.16 | *RrPMI2* | F | AGGCTTTGTCAATACAGGCTCA |
|  |  | R | CATCCTTGTAAACACTGGGGTG |
| evm.model.Contig390.17 | *RrPMI3* | F | TGCAGGGAAAGGCAGGTTAG |
|  |  | R | TAATCTTGGTGTGGGCAGGC |
| evm.model.Contig344.38 | *RrPMI4* | F | AAATCTTGCGAGGAGTTGCTG |
|  |  | R | AACTCTGTTGATTCTCCGTGGG |
| evm.model.Contig56.5 | *RrPMM1* | F | GTGGGAGGATCCAACCTTTCTAAG |
|  |  | R | CTCAACAATGTTGCCGACCAAG |
| evm.model.Contig355.102 | *RrPMM2* | F | GCCTTGTTTGATGTTGATGGAACTC |
|  |  | R | CCAGCTCCAAGTTTGTTCTTTGC |
| evm.model.Contig367.23 | *RrPMM3* | F | CTTGTGGCTCACAAAGATGGGAAG |
|  |  | R | CTTGGCTACAGTTTCGCCCAATC |
| evm.model.Contig85.109 | *RrPMM4* | F | GGTCTTGTGGCTCACAAAGATGG |
|  |  | R | CAAAGCTTATCTGTCCTCCCATGG |
| evm.model.Contig265.196 | *RrGMP1* | F | TGCCAACGAGTTTGGTGAGT |
|  |  | R | CTTCCCGGTGACTGGATACG |
| evm.model.Contig418.691 | *RrGMP2* | F | GTTAAGAGCCAGATGGGGAGC |
|  |  | R | GCCGGAATCTAGTGCCTTTAGT |
| evm.model.Contig317.289 | *RrGMP3* | F | TGGTGAACCCTTCTTTGTCCTT |
|  |  | R | ATGGCTCATCCACCTTGGTC |
| evm.model.Contig254.11 | *RrGME1* | F | ACATTTATGGACCCTTCGGCACT |
|  |  | R | CCCGTCTCCCCACATCTCAAACCT |
| evm.model.Contig250.37 | *RrGME2* | F | TCTATGGTCCTTTCGGAACCTG |
|  |  | R | CCATCTCCCCACATCTCAAACT |
| evm.model.Contig116.14 | *RrGGP1* | F | TACTGGGGAAGGGTAAACTTGG |
|  |  | R | AGGCAGAATCAGGGACATCG |
| evm.model.Contig360.207 | *RrGGP2* | F | AAGCTCCTGGCTGAGGTCTCT |
|  |  | R | CCATCATCGCCACCACAAGCAAT |
| evm.model.Contig367.80 | *RrGGP3* | F | GGGTAACACAGGCCTCAAGC |
|  |  | R | CCTTCTCCGGCAATCCATAGT |
| evm.model.Contig284.96 | *RrGPP1* | F | GCACAAACCTGGCACAAGC |
|  |  | R | AAGGGCATAAGCATAGCAGTCA |
| evm.model.Contig106.211 | *RrGPP2* | F | TCCCTCAACTCAAGGCAGCTA |
|  |  | R | TGGGCACCCACTTTCACATAC |
| evm.model.Contig296.53 | *RrGalDH1* | F | TCAAGCTCAGCTGCGTTGAC |
|  |  | R | CTCCGACAATGTCCCTCCATAG |
| evm.model.Contig231.252 | *RrGalDH2* | F | CGGGACTTCCTTTGGGAGTT |
|  |  | R | TACGCCAACACCTTTGCTCT |
| evm.model.Contig231.260 | *RrGalDH3* | F | AGTTTCCGACGAAGAAGCCATC |
|  |  | R | CGCACTTCGTTGCCACAATATAC |
| evm.model.Contig134.5 | *RrGalDH4* | F | AACGTCTTCAGCCCAGTTTCC |
|  |  | R | CTCCATAGTACGGGGAGGTGTC |
| evm.model.Contig243.49 | *RrGLDH* | F | GATTGGTGGCATTGTGCAGG |
|  |  | R | TCCCTTAGCAGGAGTGACCA |
| evm.model.Contig190.59 | *RrMIOX1* | F | TTCATTGCCCCCGACATCAA |
|  |  | R | GCTCGATTTGCGGTTCATCC |
| evm.model.Contig401.15 | *RrMIOX2* | F | ACCAGACTTATGACTTCGTCCAG |
|  |  | R | GTCCTTTCTAATGGCTTCAGCTG |
| evm.model.Contig209.44 | *RrMIOX3* | F | GCATATGGGAATGTTGTGAGCTC |
|  |  | R | TCCTTGAAGTACTTGTGGTGAACG |
| evm.model.Contig172.25 | *RrMIOX4* | F | TGGGATTGGCCTGGAGAATG |
|  |  | R | TGCTTATACGCCCCGCATTT |
| evm.model.Contig289.226 | *RrGLOase1* | F | AAGAGCATGAAGGTGGCGACTC |
|  |  | R | CTGTCATCGTCATCCCTTCAACG |
| evm.model.Contig289.225 | *RrGLOase2* | F | GGATATCAGAACCGGCTTCAAGC |
|  |  | R | ACGGACAAGCTGATGCTGAAG |
| evm.model.Contig283.191 | *RrGLOase3* | F | ACGGTGTTTGGAACGACAGA |
|  |  | R | TTTCCGCAGTCCTACACCAC |
| evm.model.Contig37.8 | *RrGalUR1* | F | CTATGCCAGTAGTAGGCATGGGAACT |
|  |  | R | AAGCAAAGGCGGTGTCAAAGTG |
| evm.model.Contig143.98 | *RrGalUR2* | F | GTGAAGAGACTGAGGGGTTTTG |
|  |  | R | GCTTTTCGCTACCATGCTCA |
| evm.model.Contig54.78 | *RrGalUR3* | F | CCTAAGCTCCTCCGGTGATG |
|  |  | R | TGATCGCTTCGAGAATGGCA |
| evm.model.Contig296.50 | *RrGalUR4* | F | AGCAATCAAAGTGGGTTACCG |
|  |  | R | GCTTGGTTGTGATGAAGAGCTCA |
| evm.model.Contig409.1 | *RrGalUR5* | F | TTTTAGGCTCACAAGTCCTCCAAC |
|  |  | R | ACCAGTCGAAGATATCAAGGTTCT |
| evm.model.Contig289.99 | *RrUBQ* | F | ATGCAGATYTTTGTGAAGAC |
|  |  | R | ACCACCACGRAGACGGAG |

**SUPPLEMENTAL TABLE 5 | List of qRT-PCR primer sequences.**
